# Pitfalls in estimating secular changes in incidence and prevalence of dementia from administrative datasets

**DOI:** 10.1002/dad2.70305

**Published:** 2026-03-11

**Authors:** Leon Flicker, Patrick Fitzgerald, Osvaldo P. Almeida, Kaarin J. Anstey, Michael Waller, Michelle Trevenen, Annette J. Dobson

**Affiliations:** ^1^ Western Australian Centre for Health and Ageing, Medical School University of Western Australia Perth Western Australia Australia; ^2^ Institute for Health Research University of Notre Dame Fremantle Western Australia Australia; ^3^ University of New South Wales UNSW Ageing Futures Institute Sydney New South Wales Australia; ^4^ School of Public Health Faculty of Health Medicine and Behavioural Sciences University of Queensland Brisbane Queensland Australia

**Keywords:** Alzheimer's disease, data linkage, dementia, incidence, prevalence, secular changes

## Abstract

**INTRODUCTION:**

Administrative datasets can be used to calculate population incidence and prevalence of dementia. It is unclear how changes in data sources may affect these estimates.

**METHODS:**

We obtained linked administrative health data for individuals 60 years of age or older in Western Australia for 1989–2019 (*n* = 893,243) including hospital admissions, emergency department, cause‐of‐death, aged care assessment (from April 2003), and mental health services.

**RESULTS:**

There was a marked increase in prevalence and incidence estimates over time. There appeared to be two phases: an initial increase attenuated by 1995–1999 and a rapid increase since 2000–2004 corresponding to inclusion of aged care assessments. There was a decrease in 2015–2019 coinciding with the unavailability of aged care assessment data.

**DISCUSSION:**

An apparent secular change in rates of dementia over 31 years may be a product of increased propensity to record dementia diagnosis and availability of additional aged care data. Consistent comprehensive data coverage is required.

## Highlights

- Administrative datasets may be a low cost method to estimate population prevalence of dementia but it is unknown whether these estimates are stable over time.
- We demonstrate over a 31‐year period that incidence and prevalence rates are prone to bias from secular trends in diagnosis and changing availability of specific data sources.
- Using administrative datasets to estimate dementia rates over time will require consistent and comprehensive data coverage.

## 1 INTRODUCTION

Determining the prevalence of dementia is essential for service planning. Unfortunately population surveys are rarely performed nowadays because of low response fractions and poor coverage of older age groups where dementia is more common.^1^ This is further complicated by the fact that registries and cohort studies have methodological shortcomings that raise concerns about the validity and generalizability of their findings.^1^ Consequently, current population estimates of the prevalence of dementia frequently rely on meta‐analyses of community studies performed several decades ago.^2^

An alternative is to use health administrative datasets, usually collected for funding purposes. The main advantage for their reuse for epidemiological purposes is the ability to obtain large amounts of data at virtually no additional costs. Their main disadvantage is the suboptimal ascertainment of cases and non‐cases.^3^ Recently, a study reported marked secular trends in dementia based on targeted surveys of U.S. Medicare claimants. The authors found a dramatic reduction in age‐specific prevalence over time,^4^ although such a finding could have been influenced by secular changes, such as variations in the construction and content of the relevant database over time.^5^ As there is a continuing need to assess the use of administrative datasets for their strengths, limitations, and biases, we attempted to replicate these findings by examining changes in dementia prevalence and incidence obtained from a large administrative database, the Western Australia Data Linkage Services [WADLS].^6^

## 2 METHODS

### 2.1 Administrative datasets and linkage

The study population consisted of individuals 60 years of age or older at any time registered on the Western Australia Electoral Roll (WAER) (compulsory in Australia) from November 1988 until December 31, 2019. Linked records were available for all inpatient public and private hospital admissions (Hospital Morbidity Data Collection [HMDC]), death registrations and causes of death (WA Register of Births, Deaths and Marriages [WA RBDM]), all public sector mental health services (WA Mental Health Information Services [WA MHIS]), the aged care assessment program (ACAP), and Health and Community Care (HACC) services. The ACAP is a nationally funded universal assessment program that allows access to subsidized home care and residential care services. Physical and cognitive health is assessed by health professionals. The number of records identified and the time periods covered are summarized in Table 1. These datasets were used to identify diagnoses of dementia from January 1m 1970 to December 31m 2019. Diagnosis codes in International Classification of Diseases, 9th Revision (ICD‐9) were converted to ICD‐10. Incidence and prevalence rates of dementia were calculated for five‐yearly age groups from 1989 to 2019, and then averaged over 5‐year periods. Data were linked across the various sources by the Data Linkage Unit of the Department of Health of WA using the unique system identifier of the HMDC as the common linkage key. Relevant demographic variables, from any source, included date of birth and sex.

**RESEARCH IN CONTEXT**

1. **Systematic review**: The authors reviewed the literature using traditional (e.g., PubMed) sources on methods for estimating the prevalence and incidence of dementia, including Alzheimer's disease. Traditional methods using specific community‐based surveys were found to be problematic due to poor population coverage including declining response fractions. Recently methods using linked administrative datasets have been developed but their use over prolonged periods have not been evaluated.
2. **Interpretation**: Our study of calculated rates derived from administrative data over a 31‐year period suggests that estimates are prone to bias from secular trends in diagnosis and changing availability of specific data sources (particularly for aged care).
3. **Future directions**: The use of administrative data sets to determine population incidence and prevalence of dementia offers a relatively low‐cost option but presents challenges. The issues of inconsistent case ascertainment will require frequent validity checks to determine any potential under or over counting of diagnoses.

**TABLE 1:** Source, number, and time period of records used in analyses, ordered by start date.

Source	*N* individuals	*N* records	Start date	End date
Hospital Morbidity Data Collection, HMDC	2,345,812	20,174,249	January 1, 1961	April 30, 2020
Death registrations, WA RBDM	434,105	434,105	April 19, 1969	September 6, 2020
WA Cancer Registry, WACR	256,500	256,500	January 4, 1982	May 24, 2020
WA Electoral Roll, WAER	1,304,041	10,567,234	November 1, 1988	June 1, 2020
Health and Community Care, HACC	161,207	2,189,443	January 1, 1990	December 12, 2020
Mental Health Information Services, MHIS	179,168	11,649,947	July 26, 2001	June 30, 2020
Emergency Department Data Collection, EDDC	974,650	5,942,956	January 15, 2002	August 15, 2020
Aged Care Assessment Program, ACAP	119,073	258,794	April 9, 2003	March 31, 2016

### 2.2 Statistical methods

Incidence and prevalence rates of dementia were calculated based on the numbers of people identified as having dementia by WADLS. From 1989 to 2019, incidence rates were calculated as the number of new dementia records that occurred within each year divided by the number of previously undiagnosed individuals alive during the same period. Prevalence rates were calculated as the number of persons living with dementia divided by the number of individuals alive during each year. The yearly incidence and prevalence rates were averaged over 5‐year periods from 1990 to 2019.

### 2.3 Comparison with other studies

The calculated prevalence was compared to population estimates from the Australian Institute of Health and Welfare (AIHW)^7^ and a worldwide meta‐analysis.^2^ The five‐yearly age‐specific incidence was compared to a large Australian study based on administrative data from the Australian state of New South Wales^8^ and a worldwide meta‐analysis.^9^

## 3 RESULTS

From November 1988 until the end of the study period, a total of 900,457 individuals 60 years of age or older or more had a recorded entry on the WAER. Of these, 6186 were listed as deceased in the WAER but were not present in the WA death registry file, suggesting that there was an error on WAER or they had died elsewhere—these individuals were excluded. An additional 1028 participants were listed as living past 114 years, suggesting that they had moved or there was an issue with their date of birth and so they too were excluded. Thus data from 893,243 individuals were included in the analyses.

Of all participants, 50.6% were female. Of the entire cohort, 98,243 (11.0%) were diagnosed with dementia. Most dementia diagnoses were first identified from HMDC admissions (54,960, or 55.9%; see Table 1), followed by HACC/ACAP assessments (36,055, 36.7%). There were 20,627 (21.0%) who were identified on HACC/ACAP assessments who were not identified on HMDC.

**FIGURE 1:**
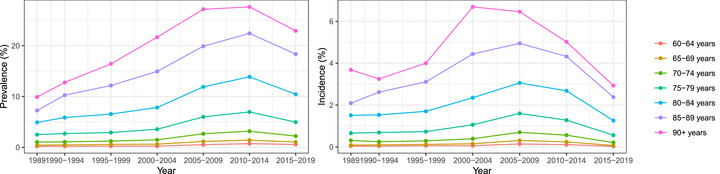
Prevalence and incidence rates by age groups over time period.

There was a marked increase in prevalence and incidence in dementia over the 1989–2014 period (Figure 1 and Appendix Tables 1 and 2 for yearly rates). This increase seemed consistent for all age groups. There appeared to be two phases of the increase in age‐specific incidence and prevalence of dementia, an initial increase that attenuated by 1995–1999, and then another phase of rapid change since 2000–2004 (Figure 1 and Appendix Figure 1).

For the last available period, 2015–2019, there was a decrease in prevalence and incidence for all age groups (Figure 1) coinciding with the unavailability of data from the ACAP.

Throughout the entire study period, the main type of dementia diagnosis available from the data sources was “dementia otherwise unspecified or not specified.” Alzheimer's dementia was by far the most common specific dementia diagnosis, greater than 70% of the total specified (Appendix Table 3).

## 4 DISCUSSION

In this study, the most striking finding was an apparent doubling of estimates of age‐specific incidence and prevalence of dementia between 1990 and 2014, suggesting two influences. First, a gradual increase in diagnoses for the period 1989–2000, possibly as a result of greater awareness of dementia by both the public and health professionals. Second, a marked increase in diagnoses after the period 2000–2004, corresponding to the availability of additional data from the Aged Care Program. Meta‐analyses have shown either stable or decreasing age‐specific prevalence rates, at least for high income countries over the period 1985 until 2011,^10^ and thus it is unlikely that we are seeing real increases in prevalence in Western Australia over this period. Rather we postulate that our estimates based on administrative sources may be affected by the source of information, access to treatment, dementia awareness campaigns and health policies. Approximately 21% of diagnoses were recorded in the aged care assessment system and not in the HMDC, reinforcing the need for incorporating as many sources of linked administrative sources as possible. The decrease in prevalence and incidence for the last period of 2015–2019 is best explained by the loss of access to the data from aged care assessments.

The results of dementia incidence for the 2010–2014 period were similar to those found in another study based on Australian administrative data.^8^ When comparing these two studies, for the age groups 60–64, 65–69, 70–74, 75–79, 80–84, 85–89, and 90+ years, our estimated incidence rates were 0.11, 0.24, 0.56, 1.28, 2.68, 4.32, and 5.41 per 100 persons/year for the period 2010–2014 (see Appendix Table 1). The comparative Australian study that included participants enrolled in 2006–2009 and followed for a period of 4.2 years, reported the respective incidence rates of 0.09, 0.22, 0.60, 1.45, 2.92, 4.84, and 7.90 per 100 persons/year.^8^ There were no apparent systematic differences between these two studies.

In comparing our prevalence data to international meta‐analysis estimates, our prevalence estimates from the period 2010–2014 for the age groups 60–64, 65–69, 70–74, 75–79, 80–84, 85–89, and 90+ years were 0.75, 1.44, 3.24, 6.98, 13.88, 22.41, and 29.18 per 100 persons (see Appendix Table 2), whereas pooled prevalence figures for the corresponding age groups were 1.8, 2.8, 4.5, 7.5, 12.5, 20.3, and 38.3 per 100 persons,^9^ suggesting that our study may have shown lower prevalence in the age groups under the age of 75 years.

There are some strengths and weaknesses in our approach. Strengths include the ability to link data for many geographically identified individuals over an extended period due to the early adoption of a unique patient identifier in Western Australia. We were also able to link to government‐funded aged care services that provided considerable additional diagnoses beyond the routine hospital admissions.^11^ Weaknesses include the lack of linkable primary care data in Australia, our inability to obtain more specific dementia diagnoses—as these were inconsistently available in the datasets, and the different temporal coverage of different data sources.

We have illustrated the potential challenges associated with the use of administrative data for determining the population prevalence of dementia. Nevertheless, the use of administrative datasets to determine population incidence and prevalence of dementia presents a relatively low‐cost option. The issues of inconsistent case ascertainment will require ongoing validity checks to determine potential under‐ or overcounting of diagnoses.

## AUTHOR CONTRIBUTIONS

**Osvaldo P. Almeida**: Conceptualization; investigation; writing—review and editing. **Kaarin J. Anstey**: Conceptualization; investigation; writing—review and editing. **Annette J. Dobson**: Conceptualization; investigation; methodology; supervision; writing—review and editing. **Patrick Fitgerald**: Data curation; formal analysis; methodology; writing—review and editing. **Leon Flicker**: Conceptualization; investigation; methodology; supervision; writing— original draft. **Michelle Trevenen**: Formal analysis; methodology; writing—review and editing. **Michael Waller**: Conceptualization; writing—review and editing.

## CONFLICT OF INTEREST STATEMENT

The authors declare no conflicts of interest. Author disclosures are available in the supporting information.

## ETHICS STATEMENT

This project was granted ethical approval by the Human Research Ethics Committee of Health Department WA.

## CONSENT

As this study utlized de‐identified data, individual consent was not necessary.

## Supporting information



Supporting infomation

Supporting infomation

## Data Availability

Data are avaiable through formal application from Data Linkage Services (DLS) of the WA Department of Health.
