# Supplementary material for: Pitfalls in estimating secular changes in incidence and prevalence of dementia from administrative datasets
Source: Alzheimers Dement (Amst). 2026 Mar 11;18(1):e70305. doi: 10.1002/dad2.70305 (PMC12976968; doi:10.1002/dad2.70305)
Supplement: Supplementary file 1 — Supporting infomation [file DAD2-18-e70305-s001.docx]

**Appendix Table 1 Incidence of dementia, per 100 persons per year, for 5-year age groups between 1989 and 2019 in Western Australia**

|  | **60-64 years** | | | **65-69 years** | | | **70-74 years** | | | **75-79 years** | | | **80-84 years** | | | **85-89 years** | | | **90+ years** | | |
| --- | --- | --- | --- | --- | --- | --- | --- | --- | --- | --- | --- | --- | --- | --- | --- | --- | --- | --- | --- | --- | --- |
|  | **F** | **M** | **All** | **F** | **M** | **All** | **F** | **M** | **All** | **F** | **M** | **All** | **F** | **M** | **All** | **F** | **M** | **All** | **F** | **M** | **All** |
| **1989** | 0.04 | 0.05 | 0.04 | 0.08 | 0.11 | 0.09 | 0.24 | 0.4 | 0.31 | 0.61 | 0.73 | 0.66 | 1.63 | 1.34 | 1.52 | 2.26 | 2.37 | 2.3 | 3.66 | 3.91 | 3.72 |
| **1990-1994** | 0.04 | 0.04 | 0.04 | 0.09 | 0.11 | 0.1 | 0.24 | 0.27 | 0.25 | 0.66 | 0.72 | 0.69 | 1.49 | 1.59 | 1.53 | 2.8 | 2.67 | 2.75 | 3.86 | 3.33 | 3.71 |
| **1995-1999** | 0.05 | 0.06 | 0.06 | 0.09 | 0.14 | 0.12 | 0.25 | 0.33 | 0.29 | 0.71 | 0.76 | 0.73 | 1.67 | 1.74 | 1.7 | 3.18 | 2.97 | 3.11 | 4.82 | 4.53 | 4.74 |
| **2000-2004** | 0.05 | 0.06 | 0.06 | 0.14 | 0.15 | 0.15 | 0.37 | 0.42 | 0.39 | 1.09 | 1.02 | 1.06 | 2.52 | 2.11 | 2.35 | 4.62 | 4.09 | 4.44 | 7.98 | 6.63 | 7.58 |
| **2005-2009** | 0.16 | 0.11 | 0.14 | 0.36 | 0.26 | 0.31 | 0.81 | 0.6 | 0.7 | 1.81 | 1.36 | 1.6 | 3.35 | 2.68 | 3.06 | 5.28 | 4.38 | 4.95 | 7.56 | 6.17 | 7.13 |
| **2010-2014** | 0.11 | 0.11 | 0.11 | 0.27 | 0.21 | 0.24 | 0.59 | 0.53 | 0.56 | 1.39 | 1.16 | 1.28 | 2.83 | 2.5 | 2.68 | 4.54 | 4.02 | 4.32 | 5.75 | 4.8 | 5.41 |
| **2015-2019** | 0.03 | 0.03 | 0.03 | 0.07 | 0.08 | 0.07 | 0.19 | 0.22 | 0.21 | 0.55 | 0.56 | 0.56 | 1.26 | 1.26 | 1.26 | 2.39 | 2.36 | 2.38 | 3.24 | 2.84 | 3.08 |
| **Wellberry** |  |  | 0.09 |  |  | 0.22 |  |  | 0.6 |  |  | 1.45 |  |  | 2.92 |  |  | 4.84 |  |  | 7.9 |
| **Prince HIC** |  |  | 0.35 |  |  | 0.59 |  |  | 1.03 |  |  | 1.87 |  |  | 3.4 |  |  | 5.96 |  |  | 12.49 |

Welberry refers to Welberry HJ, Brodaty H, Hsu B, Barbieri S, Jorm LR. Measuring dementia incidence within a cohort of 267,153 older Australians using routinely collected linked administrative data. Scientific Reports. 2020;10:8781

“HIC” refers to High Income Countries

Prince refers to Prince M, Wimo A, Guerchet M, Ali GC, Wu YT, Prina AM. World Alzheimer Report 2015. The Global Impact of Dementia: An analysis of prevalence, incidence, cost and trends. Alzheimer’s Disease International. 2015 (page 33)

**Appendix Table 2 Prevalence of dementia (percentage) for 5-year age groups between 1989 and 2019 in Western Australia**

|  | **60-64 years** | | | **65-69 years** | | | **70-74 years** | | | **75-79 years** | | | **80-84 years** | | | **85-89 years** | | | **90+ years** | | |
| --- | --- | --- | --- | --- | --- | --- | --- | --- | --- | --- | --- | --- | --- | --- | --- | --- | --- | --- | --- | --- | --- |
|  | **F** | **M** | **All** | **F** | **M** | **All** | **F** | **M** | **All** | **F** | **M** | **All** | **F** | **M** | **All** | **F** | **M** | **All** | **F** | **M** | **All** |
| **1989** | 0.2 | 0.25 | 0.23 | 0.4 | 0.5 | 0.44 | 0.95 | 1.14 | 1.04 | 2.55 | 2.44 | 2.51 | 5.12 | 4.57 | 4.91 | 8.5 | 6.77 | 7.91 | 10.46 | 8.59 | 9.98 |
| **1990-1994** | 0.22 | 0.25 | 0.24 | 0.44 | 0.54 | 0.49 | 1.01 | 1.17 | 1.08 | 2.74 | 2.67 | 2.71 | 6.16 | 5.4 | 5.87 | 11.45 | 9.36 | 10.74 | 15.59 | 10.8 | 14.3 |
| **1995-1999** | 0.24 | 0.33 | 0.28 | 0.51 | 0.68 | 0.59 | 1.16 | 1.35 | 1.25 | 2.92 | 2.92 | 2.92 | 6.97 | 5.86 | 6.55 | 13.18 | 10.14 | 12.17 | 20.52 | 14.62 | 18.9 |
| **2000-2004** | 0.25 | 0.31 | 0.28 | 0.61 | 0.71 | 0.66 | 1.4 | 1.61 | 1.5 | 3.7 | 3.41 | 3.57 | 8.58 | 6.74 | 7.84 | 16.22 | 12.42 | 14.94 | 26.13 | 18.19 | 23.94 |
| **2005-2009** | 0.62 | 0.48 | 0.55 | 1.33 | 1.06 | 1.19 | 3.01 | 2.39 | 2.7 | 6.77 | 5.16 | 6.01 | 13.53 | 9.74 | 11.91 | 22.25 | 15.81 | 19.9 | 31.95 | 22.5 | 29.23 |
| **2010-2014** | 0.86 | 0.64 | 0.75 | 1.69 | 1.21 | 1.44 | 3.69 | 2.7 | 3.2 | 8.07 | 5.79 | 6.98 | 15.85 | 11.44 | 13.88 | 25.18 | 18.22 | 22.41 | 32.46 | 22.25 | 29.18 |
| **2015-2019** | 0.71 | 0.54 | 0.63 | 1.2 | 0.93 | 1.07 | 2.59 | 1.88 | 2.23 | 5.8 | 4.1 | 4.98 | 12.16 | 8.44 | 10.46 | 21.14 | 14.51 | 18.34 | 27.34 | 17.66 | 23.86 |
| **Prince** |  |  | 1.8 |  |  | 2.8 |  |  | 4.5 |  |  | 7.5 |  |  | 12.5 |  |  | 20.3 |  |  | 38.3 |
| **AIHW** | 1.7 | 1.4 | 1.5 | 2.7 | 2.2 | 2.5 | 4.6 | 3.6 | 4.1 | 7.9 | 6.1 | 7.1 | 13.9 | 10.4 | 12.3 | 23.7 | 17.3 | 21 | 47.9 | 33.7 | 42.9 |

“Prince” refer to data from Prince M, Wimo A, Guerchet M, Ali GC, Wu YT, Prina AM. World Alzheimer Report 2015. The Global Impact of Dementia: An analysis of prevalence, incidence, cost and trends. Alzheimer’s Disease International. 2015.(Page 20)

AIHW refers to the report “Dementia in Australia” from Australian Institute of Health and Welfare Dementia in Australia. Canberra: AIHW; 2025.

**Appendix Table 3 Number of people by dementia diagnosis type and sex**

| **Diagnosis type** | **Females** | **Males** | **All** |
| --- | --- | --- | --- |
| Alzheimer's disease | 20725 (34.99%) | 12258 (31.42%) | 32983 (33.57%) |
| Vascular dementia | 3347 (5.65%) | 3667 (9.4%) | 7014 (7.14%) |
| Multiple dementia types | 423 (0.71%) | 340 (0.87%) | 763 (0.78%) |
| Dementia in Parkinson's disease | 549 (0.93%) | 1105 (2.83%) | 1654 (1.68%) |
| Frontotemporal dementia | 459 (0.77%) | 506 (1.3%) | 965 (0.98%) |
| Senile degeneration of brain, NEC^1^ | 58 (0.1%) | 55 (0.14%) | 113 (0.12%) |
| Dementia with Lewy bodies | 248 (0.42%) | 436 (1.12%) | 684 (0.7%) |
| Dementia with other unspecified feature | 222 (0.37%) | 160 (0.41%) | 382 (0.39%) |
| Dementia in other unspecified diseases | 217 (0.37%) | 177 (0.45%) | 394 (0.4%) |
| Dementia, otherwise unspecified | 25276 (42.67%) | 15829 (40.58%) | 41105 (41.84%) |
| Other dx. Codes, including drug/alcohol | 207 (0.35%) | 559 (1.43%) | 766 (0.78%) |
| Diagnosis not specified (HACC assessment) | 7503 (12.67%) | 3917 (10.04%) | 11420 (11.62%) |

^1^NEC: not elsewhere specified

**Appendix Figure 1: Prevalence and incidence rates by age, over time**
